# Vitreous expression of cytokines and growth factors in patients with diabetic retinopathy—An investigation of their expression based on clinical diabetic retinopathy grade

**DOI:** 10.1371/journal.pone.0248439

**Published:** 2021-05-19

**Authors:** Svenja Deuchler, Ralf Schubert, Pankaj Singh, Adonis Chedid, Natallia Brui, Ninel Kenikstul, Thomas Kohnen, Hanns Ackermann, Frank Koch

**Affiliations:** 1 Department of Ophthalmology, Goethe University Frankfurt, Frankfurt am Main, Germany; 2 Pneumological-immunological Laboratory, Goethe University Frankfurt, Frankfurt am Main, Germany; 3 Institute of Biostatistics, Goethe University Frankfurt, Frankfurt am Main, Germany; Massachusetts Eye & Ear Infirmary, Harvard Medical School, UNITED STATES

## Abstract

Diabetic retinopathy (DR) is an inflammatory condition that affects the posterior of the eye; yet, there are limited published data on techniques measuring the expression of growth and inflammatory factors (GIF) from the posterior segment. The purpose of the current study was two-fold: to sample the vitreous fluid from the eyes of patients with DR and assess the expression of GIF. As DR is an inflammatory disease, the second objective of this study was to determine the relationship between the status of DR and the expression of vitreous GIF. This non-randomized clinical trial was approved by BfARM for the analysis and evaluation of 12 eyes from patients with diabetic macular edema. Vitreous sampling was performed before treatment with fluocinolone acetonide and the extracted vitreous material was examined for the determination of GIF including interleukins 6 (IL-6) and 8 (IL-8), interferon gamma-inducible protein (IP-10), monocyte chemoattractant protein-1 (MCP-1), placental growth factor (PIGF), pigment epithelium-derived factor (PEDF), VEGF (vascular endothelial growth factor) and intercellular adhesion molecule (CD54). These were linearly compared with the grade of inflammation in the vitreous assessed via DR score and ART. Additionally, all eyes were grouped based on their diabetic retinopathy status. All cytokine levels, except MCP-1 and PEDF, were numerically higher in DME patients with proliferative DR than those with non-proliferative DR. DR grade was found to linearly correlate with the expression of CD54 (p = 0.02, rho = 0.64), IL-8 (p = 0.03, rho = 0.64) and PIGF (p = 0.007, rho = 0.76). A correlation was found between ART and CD54 (p = 0.02, rho = 0.66) and also between ART and IL-8 (p = 0.04, rho = 0.60). A trend was found between ART and PIGF (p = 0.08, rho 0.52). For IL-6, there appeared to be a trend with DR grade (p = 0.14, rho = 0.45) and ART (p = 0.09, rho = 0.51). Proliferative DR was shown to be associated with a significant higher expression of CD54, IL-8 and PIGF, thus suggesting that they are potentially important in defining and monitoring the effectiveness of a patients’ therapy. Vitreous probes may be helpful in deciding which therapy to administer (i.e. anti-VEGF or corticosteroid or both) based on the expression of GIF.

**Registry** EudraCT number: 2016-004488-38; DRKS-ID: DRKS00014915.

## Introduction

Diabetic retinopathy (DR) is a common microvascular complication of diabetes and remains one of the leading causes of preventable blindness in working‐age people [1]. Current literature suggests that DR is an inflammatory condition with neuro-vascular complications and it has been hypothesized that neuronal injury/dysfunction precedes clinical microvascular complications [2,3].

Yet in current practice the diagnosis of DR still involves a semi‐quantitative grading system based on the presence or absence of retinal lesions [4] and this is used to decide whether a patient has non-proliferative (NPDR) or proliferative diabetic retinopathy (PDR).

In the non-proliferative stage, there can be disruption of the internal blood-retina barrier and the progressive closure of retinal vessels. During this stage, microaneurysms, intraretinal bleeding, pearl-like veins and, in the pre-proliferative stage, intraretinal microvascular anomalies (IRMA) may also form. In contrast, PDR is characterized by the formation of irregular and severely leaking vessels that grow into the vitreous body and are associated with a significant risk of bleeding. The proliferation of vascular connective tissue and subsequent contraction can lead to tractional retinal detachment [5].

There is growing evidence to suggest that inflammation is involved in the pathogenesis of DR [3]. Indeed, growth factors, such as interleukin-6 (IL-6), have been correlated with diabetic retinopathy progression in PDR [6]. They have also been detected from the onset of diabetes through to the late-stages of retinopathy, [6] and are consistent with an increased presence of macular edema [7]. Hence there is a strong rationale for examining local factors to assess the progression of DR [3].

Takeuchi *et al* [3] reported that some growth and inflammatory factors (GIF) are identifiable and measurable in both aqueous and vitreous fluid samples, however, they are not necessarily linearly related. This implies that aqueous sampling alone does not provide a complete understanding of the extent of inflammation at the site of the disease in the posterior of the eye.

The relationship between inflammatory state and vitreous GIF remains unclear and is largely limited by the difficulty in obtaining samples from the vitreous. To address this, the current study was designed to permit the measurement of vitreous GIF and to determine the association between inflammatory state (as indicated by DR status and average retinal thickness (ART)) and vitreous GIF levels.

## Materials and methods

This monocentric non-randomized clinical pilot study was approved by the Bundesinstitut für Arzneimittel und Medizinprodukte (BfARM EudraCT-Number, 2016-004488-38) for the analysis and evaluation of 12 eyes from patients with DME and performed by the Department of Ophthalmology, Geothe-University Frankfurt/Main. 12 patient eyes were selected to be included in compliance with the sample size regulation set for this pilot study by BfARM.

Patients in our ambulatory clinic who had previously been clinically evaluated and deemed appropriate candidates for Iluvien therapy were asked if they would be interested in participating in this study. Prior to being enrolled, they decided whether or not they were willing to participate and if willing, written informed consent was obtained.

In this study, we focused on the screening (pre-intervention) characteristics of the patients before treatment. Therefore, we performed additional explanatory descriptive analyzes to describe the relationship between inflammatory state and vitreous GIF.

### Inclusion and exclusion criteria

All patients were included based on suitability to be treated with a 0.2 μg/day fluocinolone acetonide (FAc) implant. This was based on clinical judgment and decided prior to study initiation. The minimum age of participation was 18 years old. Inclusion criteria included the diagnosis of diabetic retinopathy and diabetic macular edema and either diabetes mellitus type 1 or type 2. Patients who were of childbearing potential agreed to a highly effective contraceptive method (Pearl index <1) during the study treatment prior to participation. Patients were required to have a central point thickness ≥250 and a score for best corrected visual acuity (BCVA) between 19 and 78 letters (ETDRS) in order to participate in the study. The recruitment date range was September 2018 to February 2019.

Exclusion criteria included a subtotal or complete vitrectomy (3 port pars plana vitrectomy), treatment with an anti-angiogenic agent, a partial vitrectomy with drug administration or laser coagulation within the 90 days prior to screening or treatment with a long-acting corticosteroid within the last 90 days prior to screening. In addition, patients who had undergone a cataract operation or posterior capsule opacification treatment within the past 90 days or had any other eye disease or clinically significant glaucoma evident at the time of screening were excluded. Patients with uncontrolled intraocular pressure (≥30 mmHg) were excluded.

Additional exclusion criteria included patients with uncontrolled hypertension (>160/90 mmHg (systole/diastole), poorly controlled diabetes (HbA1c >10%) or those diagnosed with an autoimmune disease. Patients were excluded if pregnant or breastfeeding during course of study, if they had participated in a clinical trial within 30 days prior to screening, and if they had any other condition that at the discretion of the investigator, was deemed to be inconsistent with participation in the trial. Finally, patients who reported drug or alcohol abuse within the 180 days prior to screening were excluded.

### Screening

**Fig 1:**
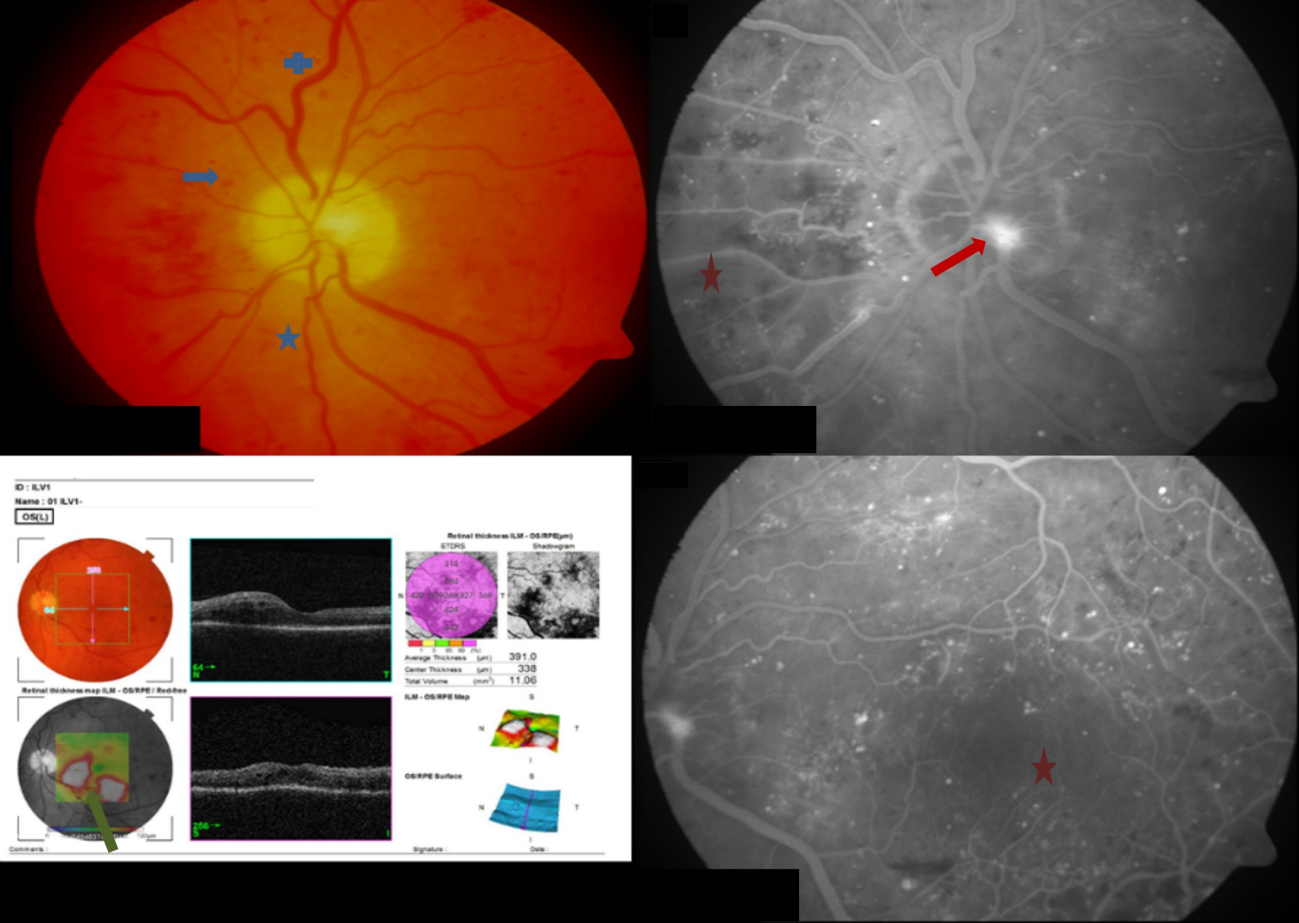
A case from a high-risk patient with PDR at screening. Image notes: (1) Top-left panel shows fundus photo and shows small dotted bleedings spots 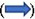 as well as curved 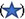 and dilated vessels 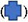. (2) Bottom-left panel shows an optical coherence tomography (OCT) image of intraretinal cysts with swelling below the fovea and in the papillomacular bundle 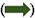. (3) Right panels are images from a fluorescein angiography and show regions of neovascularisation at the optic disc 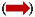 and large areas of non-perfusion 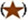 present within the nasal and macula areas (i.e. an irregular foveal avascular zone). Images also show a small zone of sub-hyaloid bleeding.

**Fig 2:**
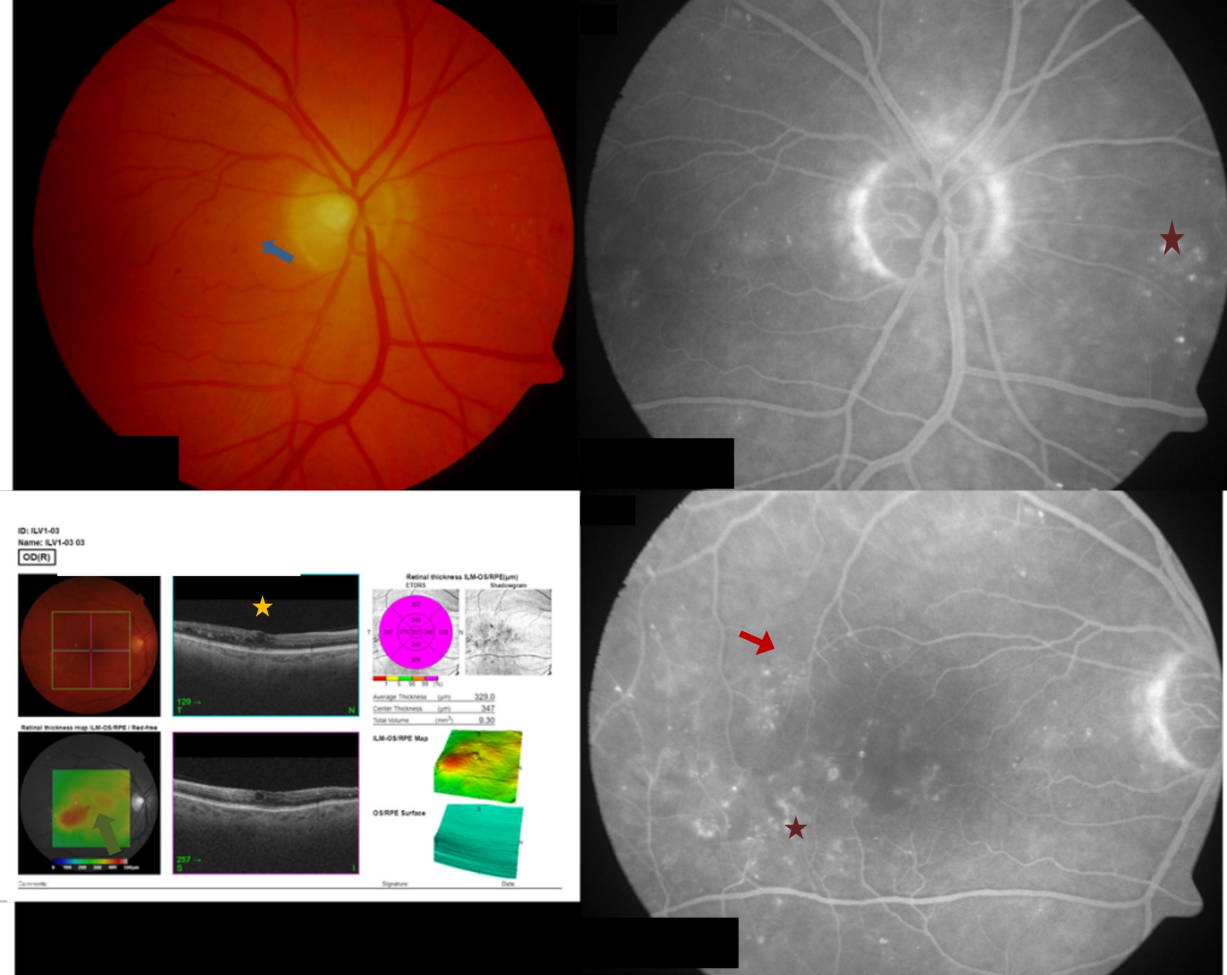
A case from a patient with moderate NPDR at screening. Image notes: (1) Top-left panel shows a fundus photo and reveals a few small dotted bleedings spots 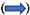. (2) Bottom-left panel shows an optical coherence tomography (OCT) image of intraretinal cysts with exudates 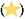 and mild edema 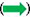 in the inferior temporal region. (3) Right panels show fluorescin angiography images from a normal optic disc, a mild intraretinal microvascular anomaly 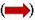 and several microaneurysms 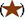.

Screening and screening data collection took place 0–4 weeks prior to baseline. At baseline, screening data were confirmed before vitreous sampling and administration of fluocinolone acetonide were performed. Data on diabetic disease status (date of first diagnosis, diabetes type, current medication, last HbA1c value) were collected. A blood sample (5–10 ml blood) was taken to determine blood sugar and HbA1c levels. Any history of diabetic macular edema (diagnosis of diabetic retinopathy, diagnosis of diabetic macular edema), prior treatments (laser, intravitreal injections, vitrectomies) and relevant concomitant diseases were recorded. Visual acuity was measured using the Early Treatment Diabetic Retinopathy Study (ETDRS) classification system. Letter score and contrast visual acuity were analyzed using Pelli-Robson Charts. Biomicroscopy under dilation was used to assess the lens and fundus. An ultrasound B image was taken to assess the vitreous state and optical coherence tomography was used to measure average retinal thickness (ART). Fluorescein angiography was utilized to assess diabetic retinopathy activity and color fundus photography was used to classify diabetic retinopathy according to ETDRS. An example of high-risk proliferative DR in comparison to moderate non-proliferative DR can be found in Figs 1 and 2.

### Vitreous sampling

**Fig 3:**
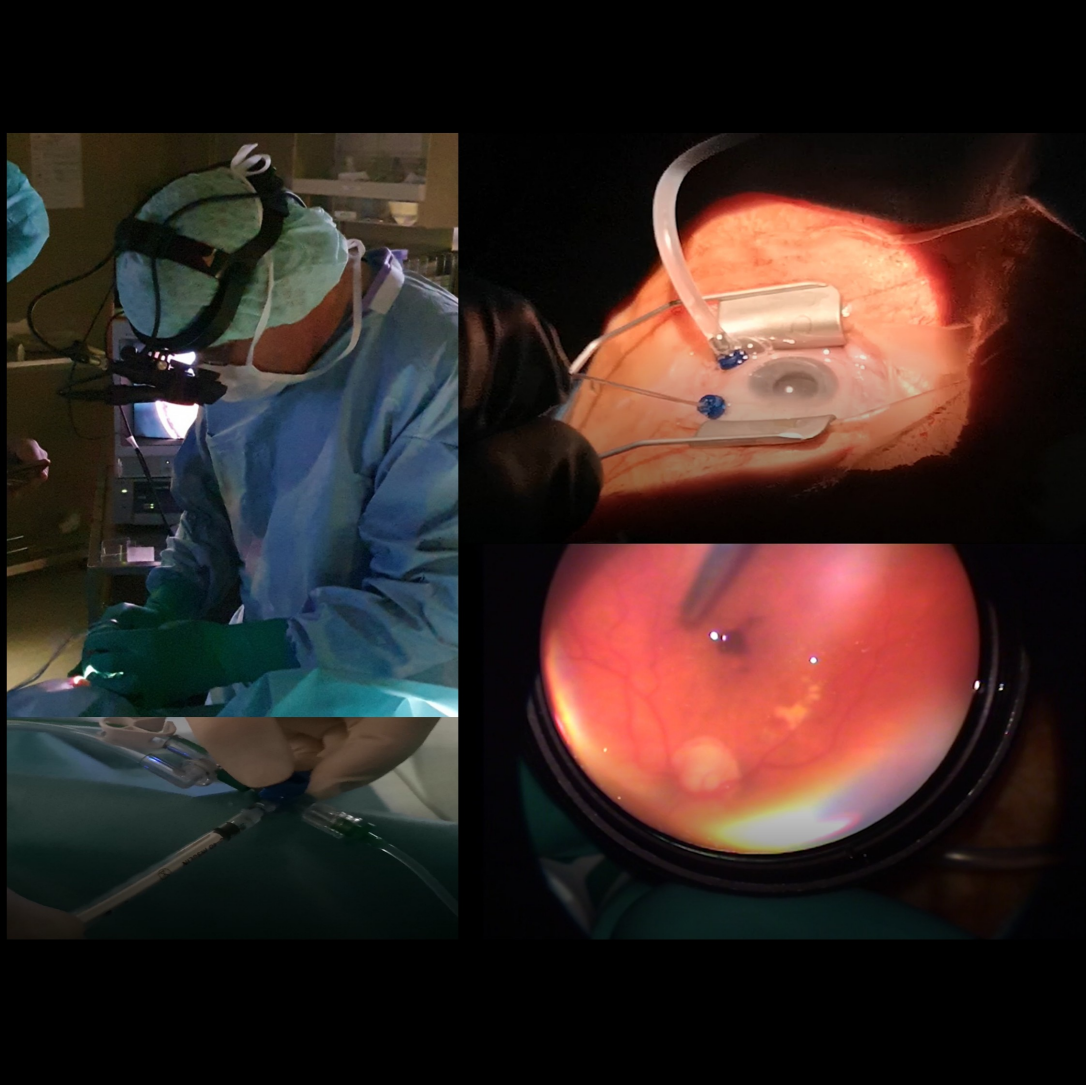
Pictures showing the vitreous sampling procedure during a partial vitrectomy. Image notes: (1) Top-left panel shows a picture of the surgeon performing a 2-port partial pars plana vitrectomy under control with the indirect ophthalmoscope. (2) Bottom-left panel shows the maneuver taking a vitreous probe. (3) Top-right panel shows an overview of the operative field. (4) Bottom-right panel shows the view through the indirect ophthalmoscope setup.

A minimally invasive 2-port partial pars plana vitrectomy was performed in all cases by one vitreoretinal surgeon in the operating room (Fig 3), before treatment with fluocinolone acetonide 190μg intravitreal. This procedure has been reported previously [8] and was associated with minimal complications (0.44%) that are comparable to the complications following the administration of intravitreal medications [8]. In brief, the procedure requires the conjunctiva to be displaced and then a vitreous probe is inserted obliquely at an angle of roughly forty-five degrees through the sclera. Once inserted, vitreous sampling can be conducted by withdrawing undiluted material from the vitreous cavity, which is then replaced with a balanced salt solution. The extracted vitreous sample was placed directly in the freezer at a temperature of -70° Celsius.

Once completed, the removal of the probe leaves a self-sealing wound, which reduces the risk of leakage from the eye and limits the penetration of pathogens from the outer ocular surface.

### Analysis of vitreous samples

Vitreous samples were sent for laboratory analysis following surgery. The lab received no information about whether each sample was part of the PDR or NPDR group. This was to allow for laboratory analysis to be conducted in a blinded fashion. Interleukin-6 (IL-6), interleukin-8 (IL-8), interferon gamma-inducible protein-10 (IP-10), monocyte chemoattractant protein-1 (MCP-1), vascular endothelial growth factor (VEGF) and intercellular adhesion molecule-1 (CD54) were measured and quantified using Cytometric Bead Array (CBA) and Flex sets [9–11]. The CBA technology is based on flow cytometric differentiation of particles (beads, 7.5 μm), which can be distinguished according to their median fluorescence intensity (MFI). For each analyte, there is a specific primary antibody. If the analyte is bound by its specific antibody then it is recognized by a second antibody to which another fluorescent dye is coupled. MFI shows the amount of bound analyte compared to a standard curve. The measuring range is between 10 pg/ml and 2500 pg/ml.

Placental Growth Factor (PIGF) and pigment epithelium-derived factor (PEDF) were detected using an Enzyme-linked Immunosorbent Assay (ELISA) (R&D Systems).

### Statistical analyses

At screening, all eyes were divided into two groups based on the presence or absence of retinal lesions. The first group (n = 7) consisted of patients with very severe non-proliferative DR (NPDR), and those with early, high-risk and severe PDR (referred to throughout as the ‘PDR group’). We decided to assign patients with very severe NPDR to this group, because 45% of patients with very severe NPDR transition to a PDR stage within one year and cytokines are detectable in the vitreous earlier than the appearance of retinal lesions [12]. The second group (n = 5) consisted of patients with mild, moderate or severe NPDR staging (referred to as the ‘NPDR group’). Eye grouping was first performed by a member of the study group and was then verified by the same experienced physician who also performed all vitreous sampling.

Fisher’s exact two-tailed tests and unpaired t-tests were performed for comparisons of patient demographics, screening values and prior treatments between the PDR and NPDR groups. Unpaired t-tests were used to assess statistical differences in VA, contrast VA, CPT, ART and GIF levels between the PDR group and NPDR group.

Vitreous inflammatory state (as assessed by the classification of DR according to ETDRS and the extent of ART) were linearly compared with vitreous GIF values using a two-sided test with Edgeworth approximation and Spearman’s Rho Correlation Coefficients.

Values are reported as means throughout unless otherwise stated and a statistically significant effect was taken as a P-value ≤0.05. All statistical analysis was performed using Microsoft Office Excel 2016 and BiAS. Version 1.12.

## Results

### Study population at screening

**Fig 4:**
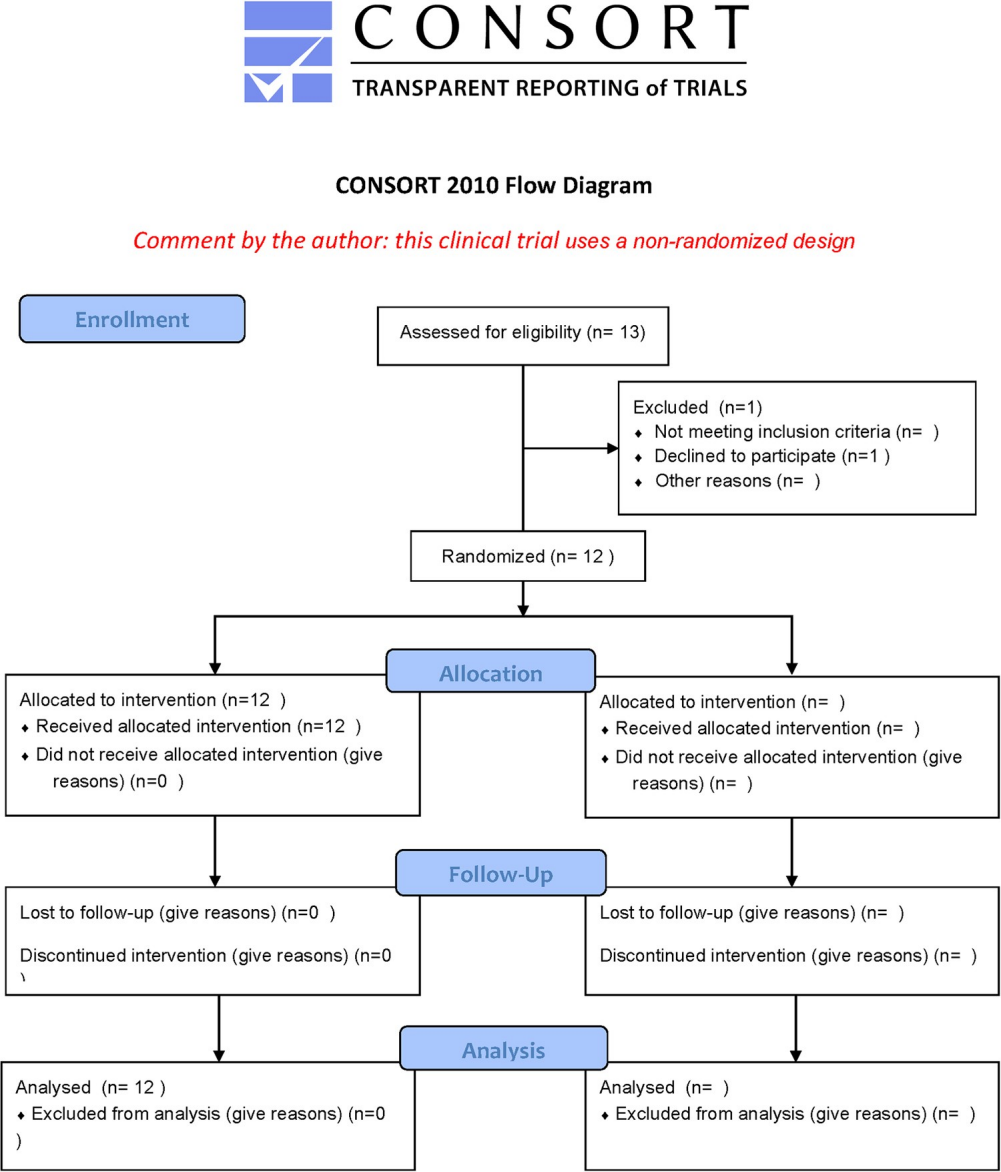
Flow chart displaying non-randomized design of study.

Out of the 13 patients assessed for eligibility, 1 patient declined to participate. 12 patients were included and received intervention (Fig 4). Data from all 12 study eyes was analyzed including data from 6 months of follow-up post injection of the FAc implant. No patients were lost to follow-up.

The mean age of treated patients was 60.92 (range, 43–78) years with the majority of patients (11 of 12) having type II diabetes which was diagnosed 13.81 (0.98–30.5) years previously. 3 of the 12 study eyes were already pseudophakic at time of screening.

Table 1 shows the patient demographics for PDR (n = 7) and NPDR (n = 5) groups and highlights that no statistically significant differences existed between groups at screening. Regarding visual acuity, both groups had similar EDTRS letter scores (67.6 vs. 66.2 [PDR vs. NPDR]; p>0.05) and contrast VA (29.7 vs. 29.2 [PDR vs. NPDR]; p>0.05).

**Table 1:** Patient demographics and screening values.

Parameters	PDR Group(n = 7)	NPDR Group (n = 5)	P-value^a^
Mean age, year (range)	57.6 (43–77)	65.6 (56–78)	0.182
Right eye/left eye, n	4/3	2/3	1.000^b^/1.000^b^
Type-II diabetes, n (%)	6/7 (85.7)	5/5 (100.0)	1.000^b^/1.000^b^
HbA1c, %	7.2 ± 1.0	6.8 ± 0.8	0.542
Mean duration since diabetes diagnosis, years (range)	11.9 (1–29.3)	16.4 (9.7–30.5)	0.447
Duration since DR diagnosis, years	1.5 ± 1.0	3.7 ± 2.9	0.164
Pseudophakic eyes, n	1	2	0.523^b^
Screening visual acuity (VA), letters	67.6 ± 10.7	66.2 ± 8.0	0.805
Screening Contrast VA, letters	29.7 ± 4.7	29.2 ± 1.3	0.792
Screening center point thickness, μm	448.9 ± 141.8	364.0 ± 72.2	0.207
Screening average retinal thickness, μm	402.1 ± 26.5	311.9 ± 34.5	0.002

Values presented as mean ± SD unless stated otherwise.

^a^ unpaired t-test for comparisons between groups.

^b^ Fisher’s exact two-tailed test unpaired t-test for comparisons between groups.

Table 2 shows the treatments received prior to the FAc implant. No statistical differences were found between the groups (P>0.05). However, in the PDR group (vs. NPDR) there numerical trends for a higher proportion of patients with a prior partial vitrectomy (57.1 vs. 20.0%; P = 0.293) for combination therapy (bevacicumab, triamcinolone and dexamethasone). Fewer were treated with prior panretinal laser (16,7 vs. 80.0, P = 0.222), therefore more patients had received prior panretinal cryocoagulation as an alternative for laser therapy in the PDR group (66.7 vs. 0.0, P = 0.081). There was no statistical difference in the number of prior anti-VEGF intravitreal injection therapies (33 vs. 45, P = 0.591), but the agent differed between groups: Only PDR patients received ranibizumab injections (28 vs. 0, P = 0.044) and the majority of NPDR patients received Aflibercept injections (5 vs 45, P = 0.137). Regarding prior corticosteroid intravitreal injections (3 vs 0, P = 0.470), dexamethasone implant injection was only given in the PDR group (3 vs. 0, P = 0.470), Triamcinolone injection was not administered in either group (P = 1.000).

**Table 2:** Prior diabetic macular edema (DME) treatments at screening.

Parameters	PDR Group (n = 7)	NPDR Group(n = 5)	P-value^b^
Prior partial vitrectomy, % (n)^a^	57.1 (4)	20.0 (1)	0.293
Laser Coagulation, % (n)	85.7 (6)	100.0 (5)	1.000
Focal laser	16.7 (1)	40.0 (1)	1.000
Panretinal laser	16.7 (1)	80.0 (3)	0.223
Focal + Panretinal laser	0 (0)	20.0 (1)	0.417
Panretinal Cryocoagulation	66.7 (4)	0 (0)	0.081
Anti-VEGF intravitreal injections, total (number of eyes)	33 injections (4)	45 injections (4)	0.591^c^ (0.579)
Ranibizumab	28 injections (4)	0 injections (0)	0.044^c^ (0.081)
Aflibercept	5 injections (2)	45 injections (4)	0.137^c^ (0.242)
Bevacizumab	0 injections (0)	0 injections (0)	1.000
Corticosteroid intravitreal injections, total (number of eyes)	3 injections (2)	0 injections (0)	0.470
Dexamethasone implant	3 injections (2)	0 injections (0)	0.470
Triamcinolone acetonide	0 injections (0)	0 injections (0)	1.000

^a^ In four of the five partial vitrectomies patients received intravitreal bevacizumab, dexamethasone and betamethasone treatments. These were not included in the anti-VEGF and corticosteroid counts.

^b^ Fisher’s exact two-tailed test unpaired t-test for comparisons between groups.

^c^ Unpaired t-test for comparisons between groups.

### Comparisons between PDR and NPDR groups

**Fig 5:**
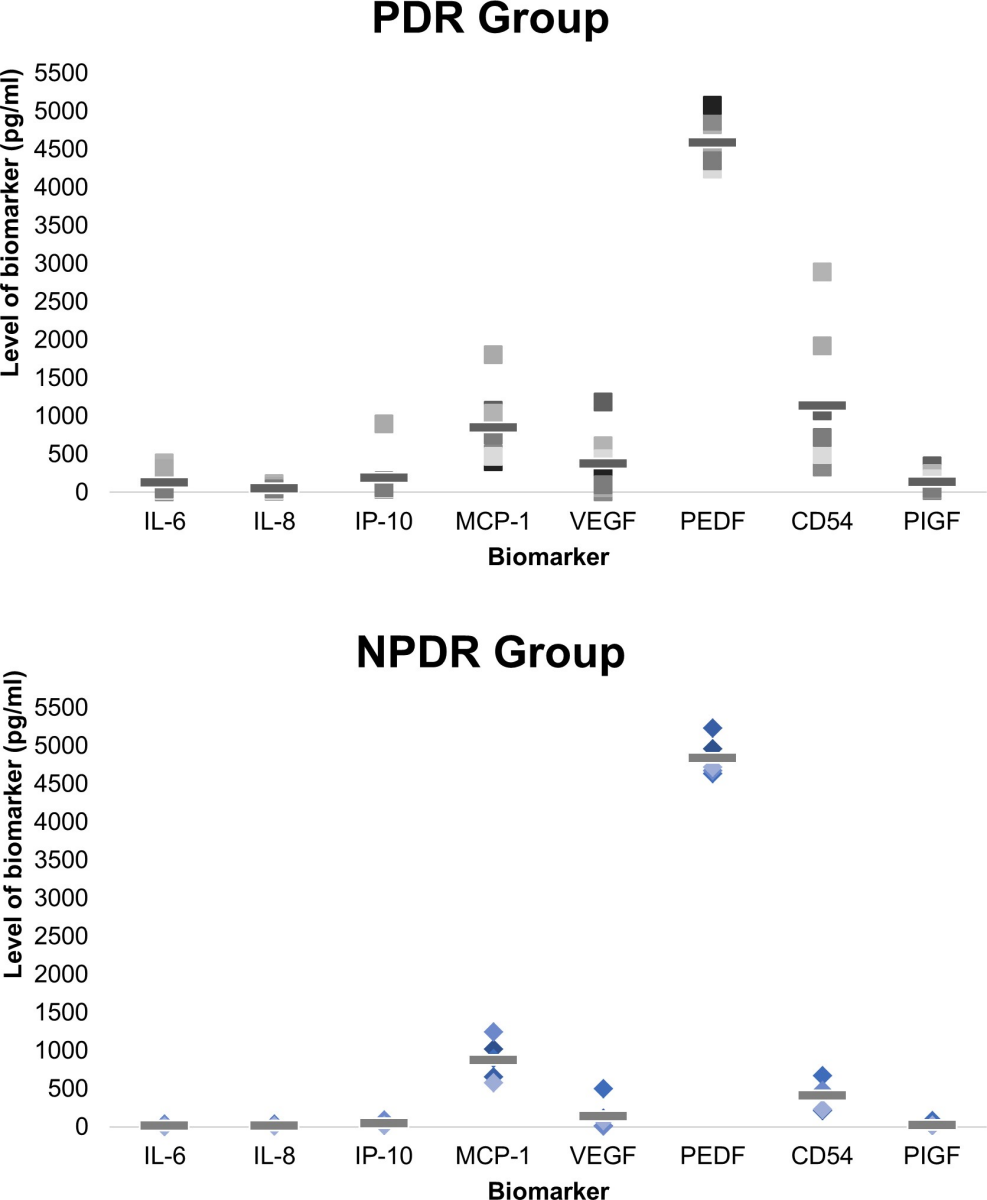
The levels of growth and inflammatory factors (GIF) in patients with proliferative diabetic retinopathy (PDR) (top panel) and non-proliferative diabetic retinopathy (NPDR) (bottom panel). Image notes: Individual data plotted using different shades of black (top panel) or blue (bottom panel) with the mean value shown as a solid horizontal line.

Fig 5 compares the mean cytokine levels in PDR and NPDR groups and shows that except for MCP-1 (858.9 vs. 877.0 pg/ml; P = 0.937) and PEDF (4,592.1 vs. 4,841.9 pg/ml; P = 0.170), the levels of IL-6 (133.3 vs. 15.8 pg/ml; p = 0.085), IL-8 (56.9 vs. 16.1 pg/ml; P = 0.029), IP-10 (194.0 vs. 51.9 pg/ml; P = 0.274), VEGF (378.6 vs. 142.6 pg/ml; P = 0.224), CD54 (1,144.9 vs. 411.6 pg/ml; P = 0.085) and PIGF (144.2 vs. 28.1 pg/ml; P = 0.044) were numerically higher in DME patients with PDR than those with NPDR with statistically significant higher values for IL-8 and PIGF observed in the PDR group. Higher values were also observed for center point thickness (CPT) and average retinal thickness (ART) in the PDR group: mean CPT, 448.9 vs. 364.0 μm (P = 0.207), PDR vs. NPDR group; and mean ART, 402.1 vs. 311.9 μm (P = 0.002), respectively.

### Correlations between vitreous inflammatory state (DR and ART) and GIFs

Vitreous inflammatory state was correlated with GIFs. DR state was found to linearly and statistically correlate with the expression of CD54 (Rho = 0.64; P = 0.02), IL-8 (Rho = 0.64, P = 0.03) and PIGF (Rho = 0.76, P = 0.007). There was a trend between DR state and IL-6 (Rho = 0.45, P = 0.14), VEGF (Rho = 0.52, P = 0.08) and PEDF (Rho = -0.54; P = 0.07). No statistically significant (P>0.05) correlations were found for other comparisons.

The same comparisons were conducted between ART and the GIFs. ART was shown to be linearly and significantly related to CD54 (Rho = 0.66, P = 0.02) and to IL-8 (Rho = 0.60, P = 0.04).There was a trend between ART and IL-6 (Rho = 0.51, P = 0.09) and a trend between ART and PIGF (Rho = 0.52, P = 0.08). No correlation was seen with the other GIFs (P>0.05), including VEGF (Rho = 0.24, P = 0.46).

### Study patient samples

Fig 1 shows images (fundus photo, fluorescein angiography and optical coherence tomography [OCT]) from a 49-year-old type II diabetic patient with high risk PDR at the time of screening. In May 2018 the patient underwent a partial PPV and received a panretinal cryocoagulation. No intravitreal injections were administered before the FAc implant was given in September 2018. At screening, visual acuity (VA) was 77 ETDRS letters, ART was 391.0 μm and GIF levels at baseline were: IL-6, 133.4 pg/ml; IL-8, 97.1 pg/ml; IP-10, 149.3 pg/ml; MCP-1, 1069.4 pg/ml; VEGF, 1178.4; CD54, 1064.7 pg/ml; PIGF, 343.4 pg/ml; and, PEDF 4,260.7 pg/ml.

In contrast, Fig 2 shows the same images but from a 56-year-old type II diabetic patient with moderate NPDR at screening. In May 2018 the patient underwent a partial PPV. In September 2010 the patient received a panretinal photocoagulation laser in the study eye. Other therapies included 5 intravitreal injections of aflibercept before the FAc implant was administered in September 2018. At screening, VA was 75 ETDRS letters, ART was 329.0 μm. At baselineGIF levels were: IL-6, 14.2 pg/ml; IL-8, 10.1 pg/ml; IP-10, 17.7 pg/ml; MCP-1, 655.1 pg/ml; VEGF, 109.0; CD54, 213.3 pg/ml; PIGF, 17.5 pg/ml; and, PEDF 5,233.0 pg/ml.

## Discussion

The current study explored the relationship between DR status and GIFs sampled from the vitreous humor of twelve patients. In the first part of the analysis, patients were grouped based on their grade of inflammation in the vitreous, assessed via DR score and further evaluated using ART. Comparison of mean values revealed that PDR, relative to NPDR, was associated with statistically higher levels for IL-8 and PIGF and that consistently higher values were reported for IL-6, IP-10, VEGF and CD54. In the second part of the analysis, linear comparisons showed that DR state statistically correlated with IL-8, PIGF and CD54 levels and trends were observed between DR state and IL-6, VEGF and PEDF. Findings suggest that the expression of IL-8, PIGF and CD54 may be important in defining and monitoring the effectiveness of a patients’ therapy. Current data suggest that vitreous probes may help define the expression of GIFs and indicate which treatment to administer.

The potential limitations of this study are the small sample size with a cohort of 12 patients and the monocentric design. From there the study needs to be confirmed with a larger sample size across multiple centers, and a focus on blinded sample collection and group assignment.

Past studies have reported the changes in GIFs that have been sampled from the aqueous or vitreous of the eye and have been summarized by Rübsam et al. [13]. A recent study by Song et al. [14] quantified the aqueous humor concentrations of IL-6, IL-8, IL-10, VEGF, TGF-β, VCAM-1, CD54, MCP-1 and showed higher levels for all parameters in patients with PDR. In contrast to this study and others, the current study is unique because it focuses on the expression of GIFs in DR patients with chronic DME (DME that persists or recurs despite prior treatment) and it reports GIFs sampled from the vitreous of the eye. Similarly to prior studies [13–17], we found elevated levels of the GIFs measured in the PDR group that are also consistent with a thicker macula (in both CPT and ART) in this group. The only two exceptions were MCP-1 and PEDF, which tended to be lower in the PDR group than the NPDR group. The reduction in MCP-1 contradicts findings by Song et al. [14] where aqueous samples were compared and mean values were shown to be increased in PDR versus NPDR. Further, in the case of PEDF, Rübsam et al. documented that patients with active PDR had increased vitreous PEDF, and in those with inactive PDR, decreased PEDF. PEDF was also decreased in patients with DME. It is unclear why this occurs in the later stages of the disease [13]; however, it has been observed in patients with hyperfluorescent DME [18,19].

Interestingly, there was only a trend, not significance, between the DR state and VEGF. This also reflects the results of earlier studies showing that VEGF is particularly useful in the earlier stages of diabetic retinopathies. As the disease progresses, VEGF decreases and other cytokines increase. For this reason, it is also useful to treat patients who are refractory to anti-VEGF therapy with steroids in clinical practice [20]. The same applies to advanced diabetic retinopathies. In this case, however, it is always necessary to be aware of the side effects of corticosteroids such as increased intraocular pressure and progression of lens opacity [12].

A number of studies have assessed the relationship between cytokines and other parameters including disease status, visual acuity and OCT parameters [14–16,18]. Funatsu et al. [18] reported the vitreous concentrations of VEGF, CD54, IL-6, MCP-1, and PEDF correlated with retinal thickness and the authors concluded that VEGF and CD54 had the strongest influence on the severity of DME. The current findings complement those findings and show a positive linear relationship between DR and IL-8, CD54, and PIGF. Trends were observed between DR status and VEGF (P = 0.08) and PEDF (P = 0.07) Other studies have considered the relationship between OCT parameters and cytokines. Funatsu et al. [18] correlated vitreous cytokines with retinal thickness and showed positive linear relationships with IL-6, MCP-1, VEGF, CD54 and PEDF. Kim et al. [16] found positive linear correlations between central foveal thickness and aqueous IL-6 and IL-8 concentrations in DME patients with cystoid macular edema. Hillier et al. [15] looked at the relationship between cytokines and central macular thickness and reported no significant correlations for central macular thickness. Positive relationships were found, however, for macular volume and aqueous IL-6, IL-8, CD54, PIGF and IL-10. The current findings clearly support the relationship with CD54 (both for correlations with DR status and ART) and support past studies that suggest it plays a significant role in DME [21]. IL-8 was also shown to relate to ART (P = 0.04) and again complements previous findings in this area [13].

The current findings complement observations that proliferative vitreoretinopathies are generally associated with increased cytokine levels [22] and for this reason their treatment represents a challenge for treating physicians. In most cases, a combination therapy with laser coagulation, anti-VEGF and intravitreal corticosteroid treatment is adopted to try and halt further progression of the disease [23–25].

Severe non-proliferative retinopathy is a direct precursor of proliferative retinopathy and cytokine levels can be detected in serum during this phase of the disease [22]. However, early forms of diabetic retinopathy do not show elevated serum levels and so vitreous samples become quite important in the choice of therapy. The current study shows the importance of taking samples from the structure close to the retina affected by the disease. Inflammation is pivotal to both neuronal and vascular changes in patients with diabetes [26–28] and so vitreous samples taken from patients with DME that persists or recurs despite treatment is helpful in defining the extent of inflammation.

The use of corticosteroids in the eye are commonly associated with the acceleration of cataract formation in patients with diabetes [29] and elevated intraocular pressure [30], and it is important to weigh these adverse events against the benefit of long-term therapy and the long-term control of the health of the macula.

## Conclusions

Our data shows that in patients diagnosed with both DR and DME, those with PDR have significantly higher levels of IL-8 and PIGF in the vitreous fluid compared to those with NPDR. Consistently higher values were also reported in the PDR group for IL-6, IP-10, VEGF, CD54, and PIGF.

Findings suggest that probing the vitreous to sample GIFs could be used as a guide to define the extent of disease progression and aid in the selection of a therapy to target the extent of localized inflammation in the eye.

## Supporting information

S1 Checklist TREND statement checklist. (PDF)

S1 File Patient screening data and OCT images. (PDF)

S2 File ELISA kits and flex sets details. (PDF)

S1 Dataset (XLSX)
