# Supplementary material for: Vitreous expression of cytokines and growth factors in patients with diabetic retinopathy—An investigation of their expression based on clinical diabetic retinopathy grade
Source: PLoS One. 2021 May 19;16(5):e0248439. doi: 10.1371/journal.pone.0248439 (PMC8133486; doi:10.1371/journal.pone.0248439)

Patient 1 (ILV-01)

**Age and sex:**	49, male
**Study eye:**	Left
**HbA1C:**	5.58
**Blood Pressure:**	140/70
**Diabetes mellitus type:**	Type 2
**Diabetes duration since diagnosis:**	1.06 years
**Taking insulin:**	Yes
**ETDRS classification of diabetic retinopathy:**	High-risk PDR
**Assigned group:**	PDR
**Fluoresceine Angiography:**	
**Leakage (Fovea centralis affected)**	Yes
**Central Ischemia:**	Yes
**Neovascularization of the optic disc (NVD):**	Yes
**Neovascularization elsewhere (NVE):**	Yes

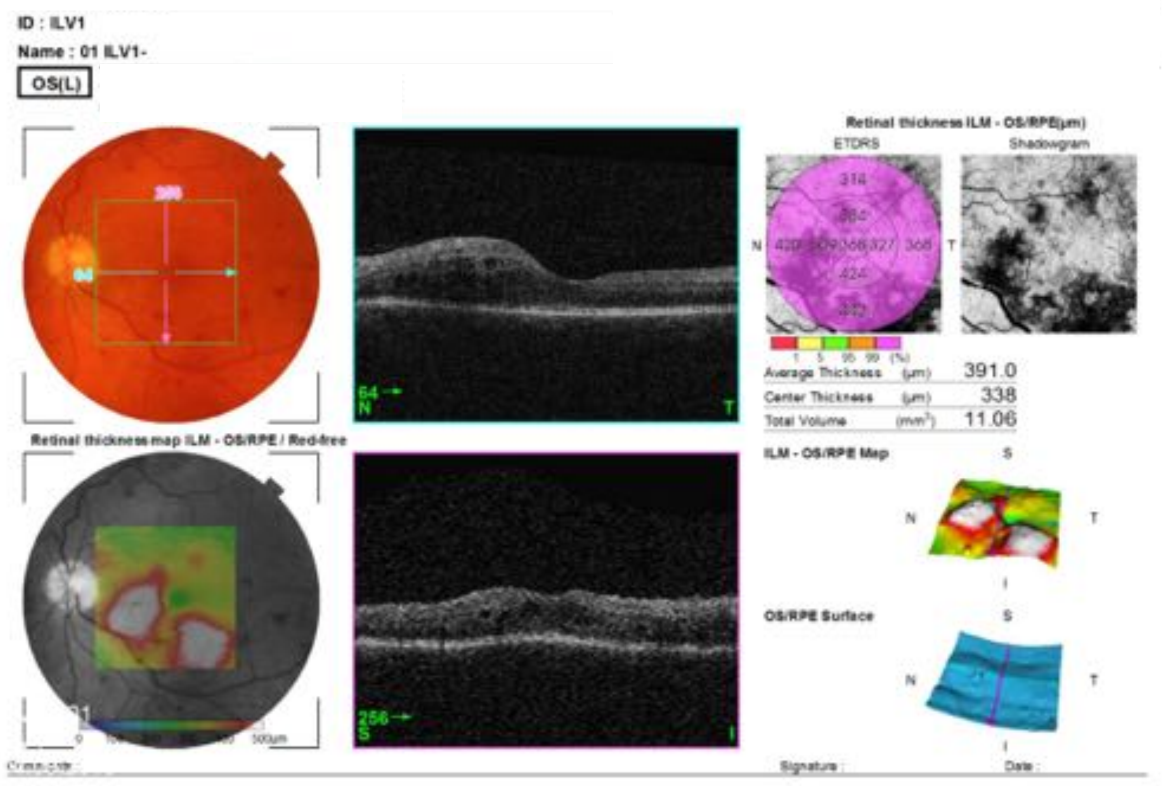

Patient 2 (ILV-02)

**Age and sex:**	60, male
**Study eye:**	Left
**HbA1C:**	5.94
**Blood Pressure:**	120/80
**Diabetes mellitus type:**	Type 2
**Diabetes duration since diagnosis:**	13.22 years
**Taking insulin:**	Yes
**ETDRS classification of diabetic retinopathy:**	Severe NPDR
**Assigned group:**	NPDR
**Fluoresceine Angiography:**	
**Leakage (Fovea centralis affected)**	Yes
**Central Ischemia:**	Yes
**Neovascularization of the optic disc (NVD):**	No
**Neovascularization elsewhere (NVE):**	No

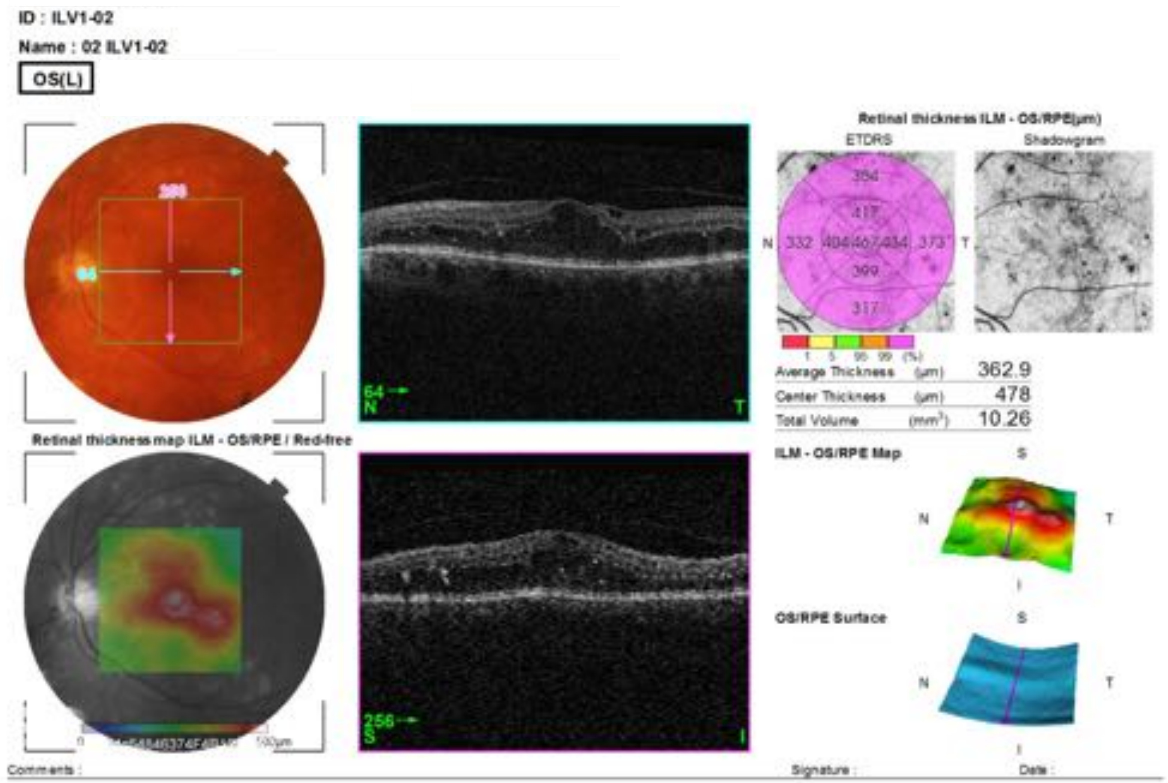

Patient 3 (ILV-03)

**Age and sex:**	56, male
**Study eye:**	Right
**HbA1C:**	6.48
**Blood Pressure:**	120/80
**Diabetes mellitus type:**	Type 2
**Diabetes duration since diagnosis:**	9.73 years
**Taking insulin:**	No
**ETDRS classification of diabetic retinopathy:**	Moderate NPDR
**Assigned group:**	NPDR
**Fluoresceine Angiography:**	
**Leakage (Fovea centralis affected)**	Yes
**Central Ischemia:**	No
**Neovascularization of the optic disc (NVD):**	No
**Neovascularization elsewhere (NVE):**	No

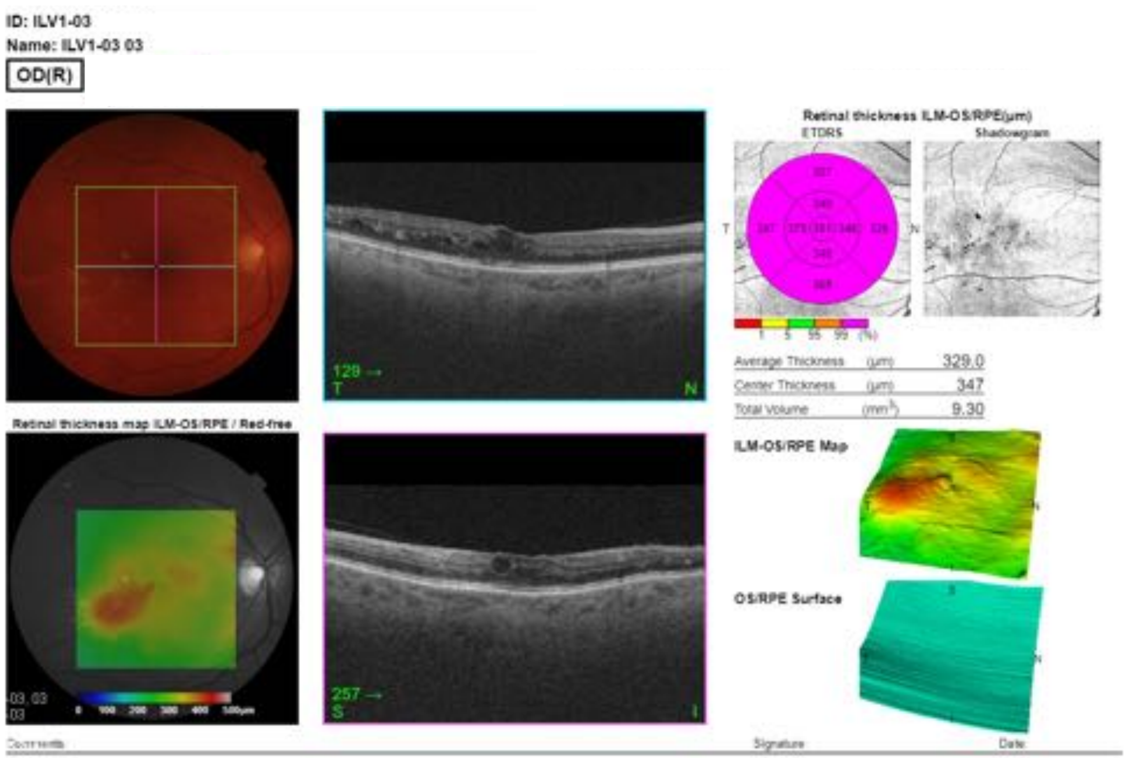

Patient 4 (ILV-04)

**Age and sex:**	78, male
**Study eye:**	Left
**HbA1C:**	6.61
**Blood Pressure:**	120/70
**Diabetes mellitus type:**	Type 2
**Diabetes duration since diagnosis:**	10.24 years
**Taking insulin:**	Yes
**ETDRS classification of diabetic retinopathy:**	Severe NPDR
**Assigned group:**	NPDR
**Fluoresceine Angiography:**	
**Leakage (Fovea centralis affected)**	Yes
**Central Ischemia:**	Yes
**Neovascularization of the optic disc (NVD):**	No
**Neovascularization elsewhere (NVE):**	No

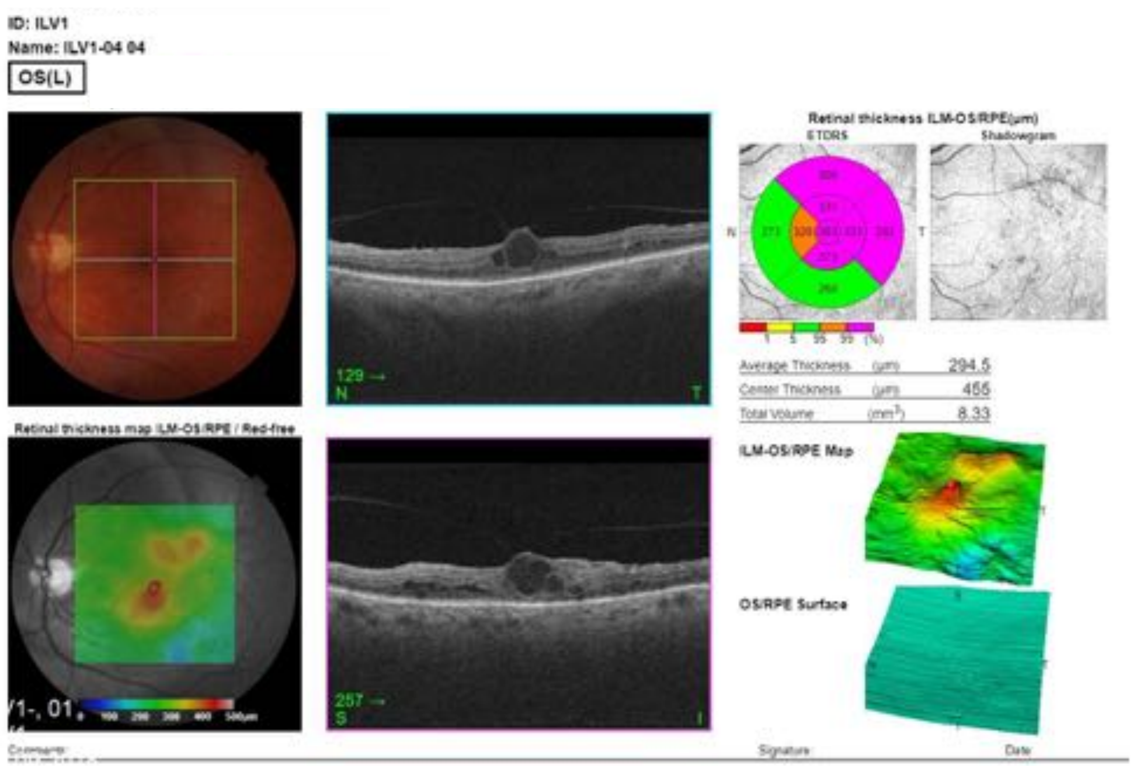

Patient 5 (ILV-05)

**Age and sex:**	59, male
**Study eye:**	Right
**HbA1C:**	8.52
**Blood Pressure:**	140/70
**Diabetes mellitus type:**	Type 2
**Diabetes duration since diagnosis:**	29.34 years
**Taking insulin:**	Yes
**ETDRS classification of diabetic retinopathy:**	Mild-moderate PDR
**Assigned group:**	PDR
**Fluoresceine Angiography:**	
**Leakage (Fovea centralis affected)**	Yes
**Central Ischemia:**	Yes
**Neovascularization of the optic disc (NVD):**	Yes
**Neovascularization elsewhere (NVE):**	Yes

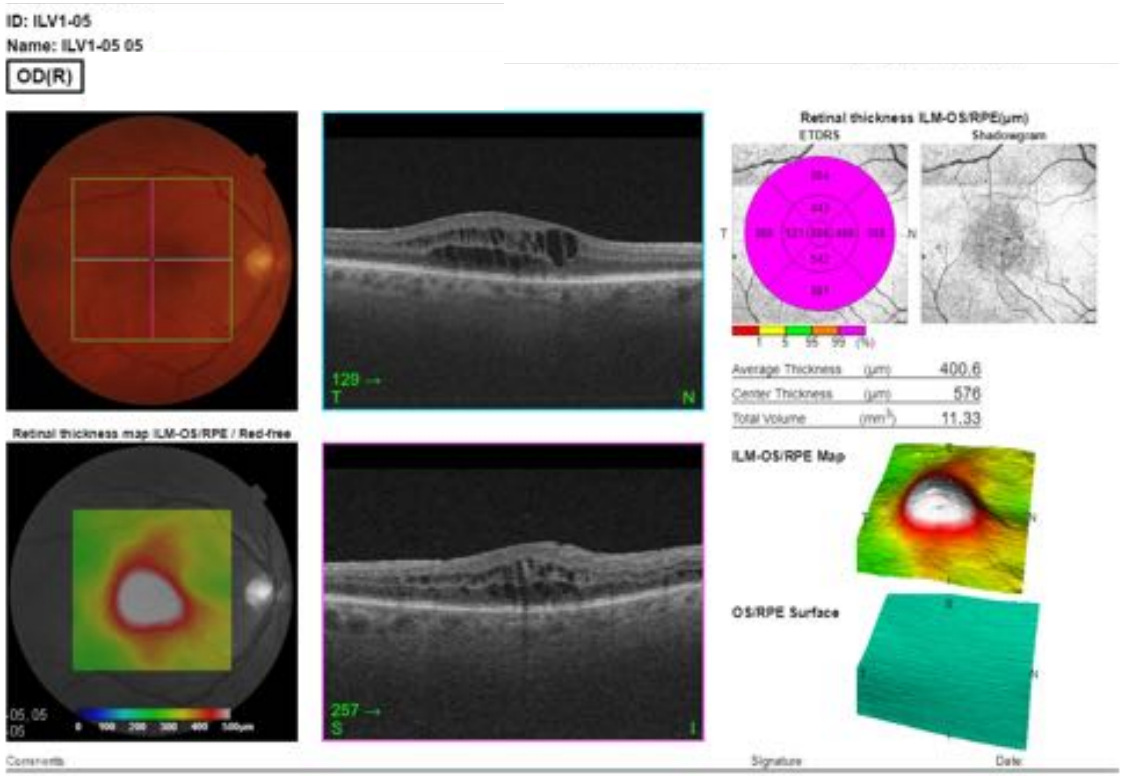

Patient 6 (ILV-06)

**Age and sex:**	60, male
**Study eye:**	Left
**HbA1C:**	7.95
**Blood Pressure:**	160/85
**Diabetes mellitus type:**	Type 2
**Diabetes duration since diagnosis:**	10.38 years
**Taking insulin:**	Yes
**ETDRS classification of diabetic retinopathy:**	Very severe NPDR
**Assigned group:**	PDR
**Fluoresceine Angiography:**	
**Leakage (Fovea centralis affected)**	Yes
**Central Ischemia:**	Not definable
**Neovascularization of the optic disc (NVD):**	No
**Neovascularization elsewhere (NVE):**	No

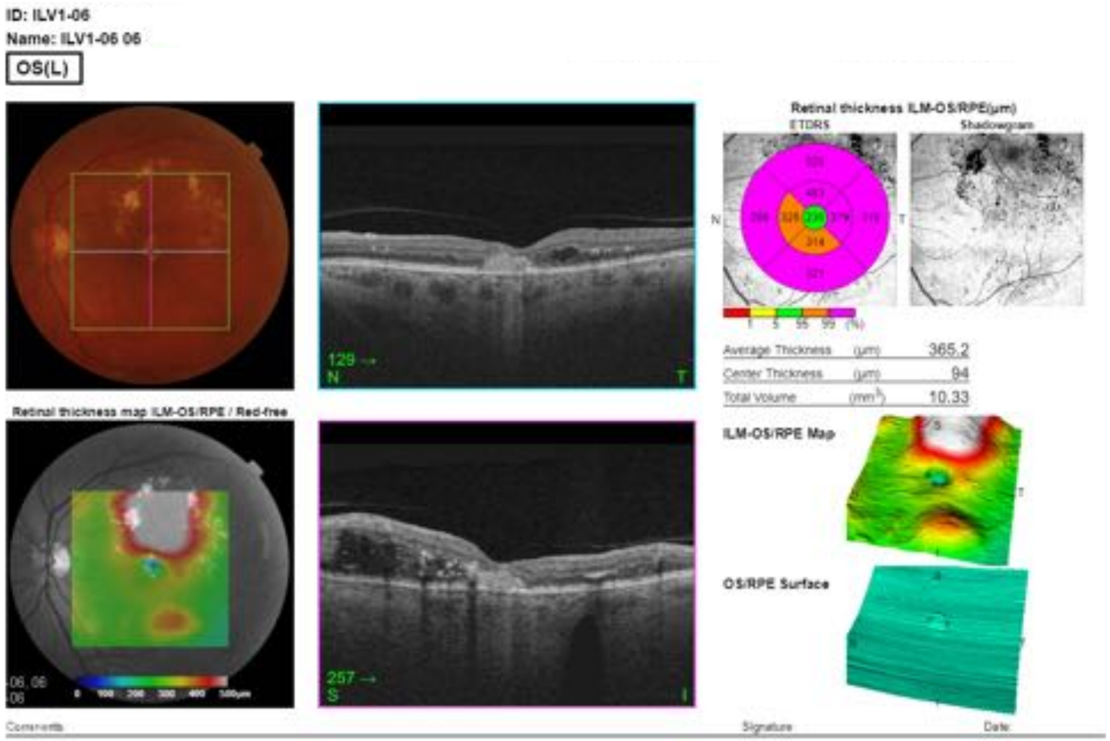

Patient 7 (ILV-07)

**Age and sex:**	70, female
**Study eye:**	Left
**HbA1C:**	7.08
**Blood Pressure:**	150/85
**Diabetes mellitus type:**	Type 2
**Diabetes duration since diagnosis:**	30.47 years
**Taking insulin:**	Yes
**ETDRS classification of diabetic retinopathy:**	Moderate NPDR
**Assigned group:**	NPDR
**Fluoresceine Angiography:**	
**Leakage (Fovea centralis affected)**	Yes
**Central Ischemia:**	No
**Neovascularization of the optic disc (NVD):**	No
**Neovascularization elsewhere (NVE):**	No

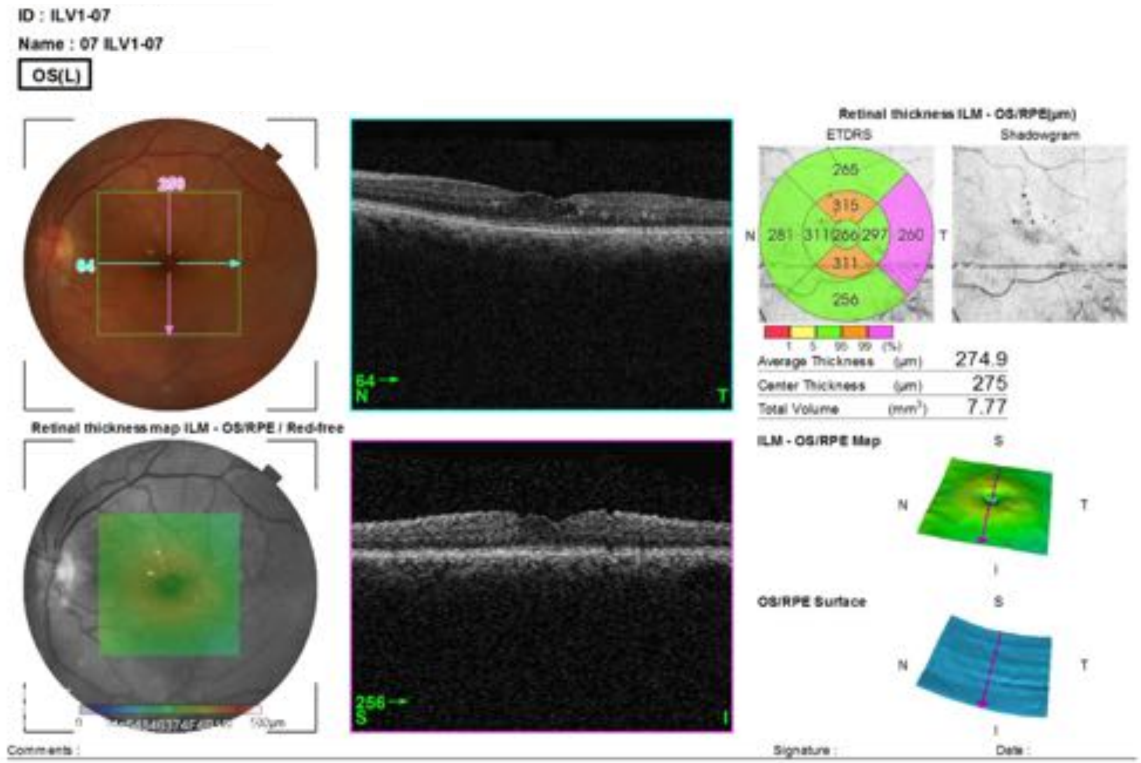

Patient 8 (ILV-08)

**Age and sex:**	55, female
**Study eye:**	Right
**HbA1C:**	6.7
**Blood Pressure:**	160/80
**Diabetes mellitus type:**	Type 2
**Diabetes duration since diagnosis:**	0.98 years
**Taking insulin:**	Yes
**ETDRS classification of diabetic retinopathy:**	Very severe NPDR
**Assigned group:**	PDR
**Fluoresceine Angiography:**	
**Leakage (Fovea centralis affected)**	Yes
**Central Ischemia:**	Yes
**Neovascularization of the optic disc (NVD):**	No
**Neovascularization elsewhere (NVE):**	No

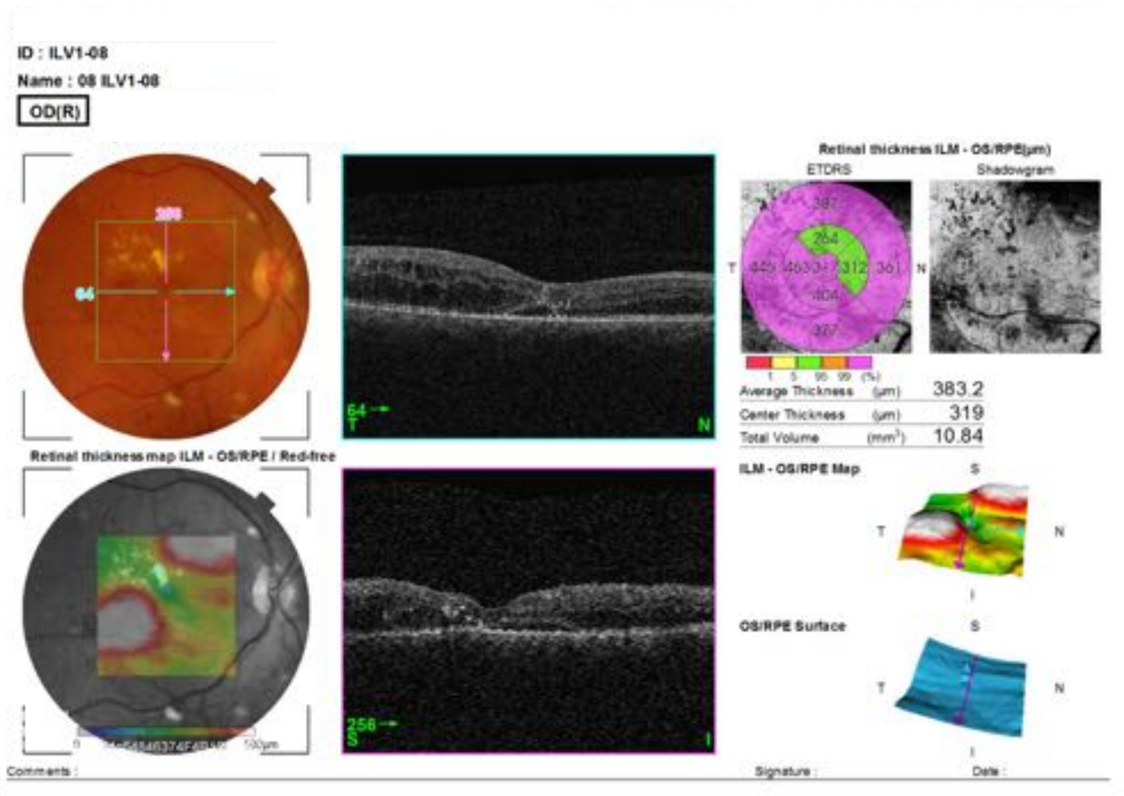

Patient 9 (ILV-09) declined to participate

Patient 10 (ILV-10)

**Age and sex:**	43, male
**Study eye:**	Right
**HbA1C:**	7.19
**Blood Pressure:**	120/90
**Diabetes mellitus type:**	Type 1
**Diabetes duration since diagnosis:**	20.54 years
**Taking insulin:**	Yes
**ETDRS classification of diabetic retinopathy:**	High-risk PDR
**Assigned group:**	PDR
**Fluoresceine Angiography:**	
**Leakage (Fovea centralis affected)**	Yes
**Central Ischemia:**	Yes
**Neovascularization of the optic disc (NVD):**	No
**Neovascularization elsewhere (NVE):**	Yes

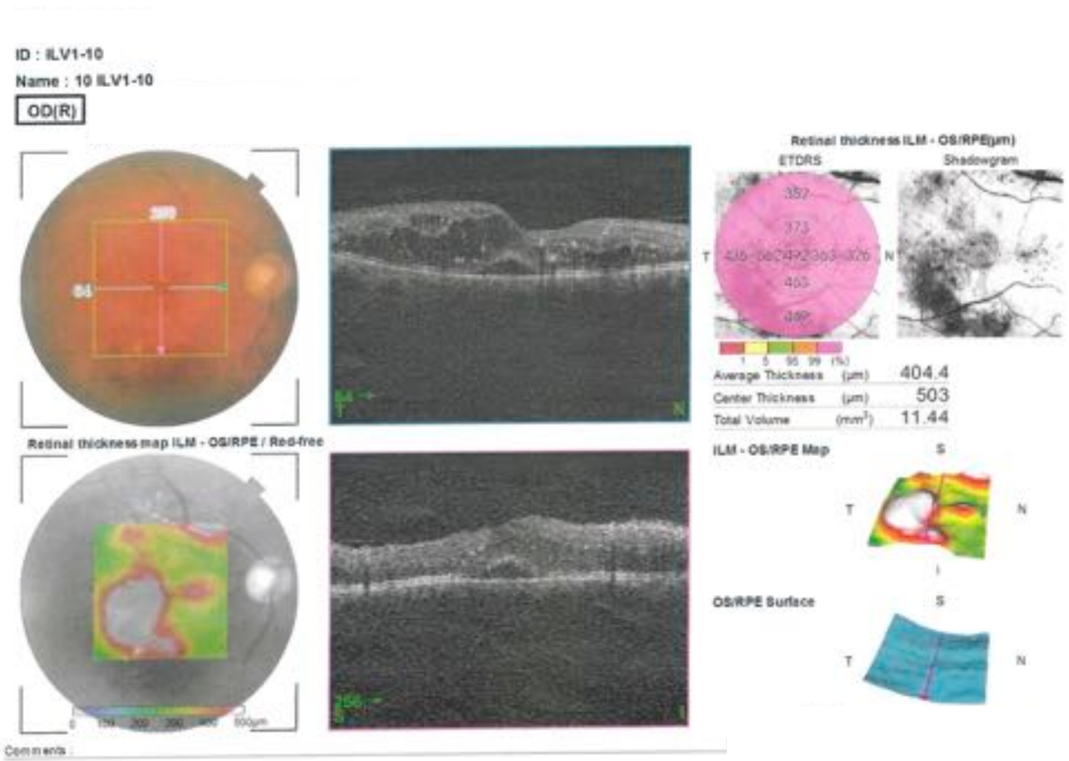

Patient 11 (ILV-11)

**Age and sex:**	77, male
**Study eye:**	Right
**HbA1C:**	7.72
**Blood Pressure:**	130/70
**Diabetes mellitus type:**	Type 2
**Diabetes duration since diagnosis:**	18.56 years
**Taking insulin:**	Yes
**ETDRS classification of diabetic retinopathy:**	Very severe NPDR
**Assigned group:**	PDR
**Fluoresceine Angiography:**	
**Leakage (Fovea centralis affected)**	Yes
**Central Ischemia:**	Yes
**Neovascularization of the optic disc (NVD):**	No
**Neovascularization elsewhere (NVE):**	No

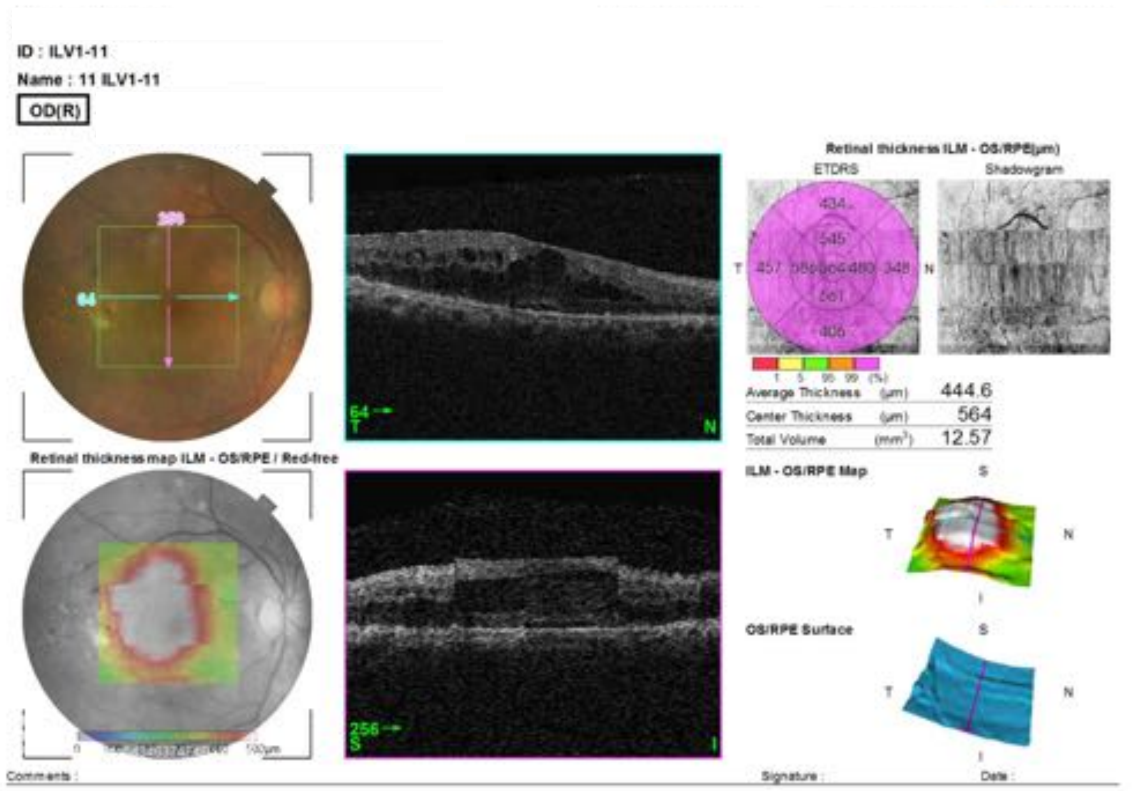

Patient 12 (ILV-12)

**Age and sex:**	64, male
**Study eye:**	Right
**HbA1C:**	8.06
**Blood Pressure:**	140/80
**Diabetes mellitus type:**	Type 2
**Diabetes duration since diagnosis:**	18.57 years
**Taking insulin:**	Yes
**ETDRS classification of diabetic retinopathy:**	Severe NPDR
**Assigned group:**	NPDR
**Fluoresceine Angiography:**	
**Leakage (Fovea centralis affected)**	Yes
**Central Ischemia:**	Yes
**Neovascularization of the optic disc (NVD):**	No
**Neovascularization elsewhere (NVE):**	No

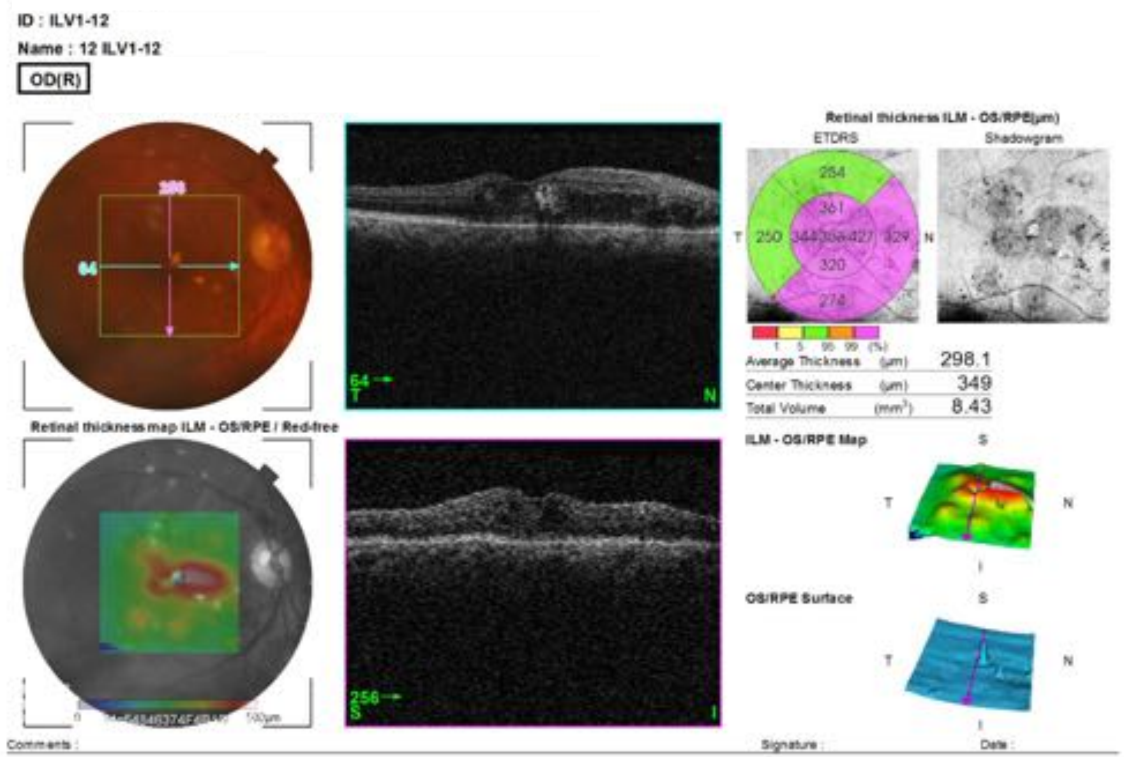

Patient 13 (ILV-13)

**Age and sex:**	60, female
**Study eye:**	Left
**HbA1C:**	6.47
**Blood Pressure:**	160/80
**Diabetes mellitus type:**	Type 2
**Diabetes duration since diagnosis:**	2.59 years
**Taking insulin:**	No
**ETDRS classification of diabetic retinopathy:**	Very severe NPDR
**Assigned group:**	PDR
**Fluoresceine Angiography:**	
**Leakage (Fovea centralis affected)**	Yes
**Central Ischemia:**	Yes
**Neovascularization of the optic disc (NVD):**	No
**Neovascularization elsewhere (NVE):**	Not definable

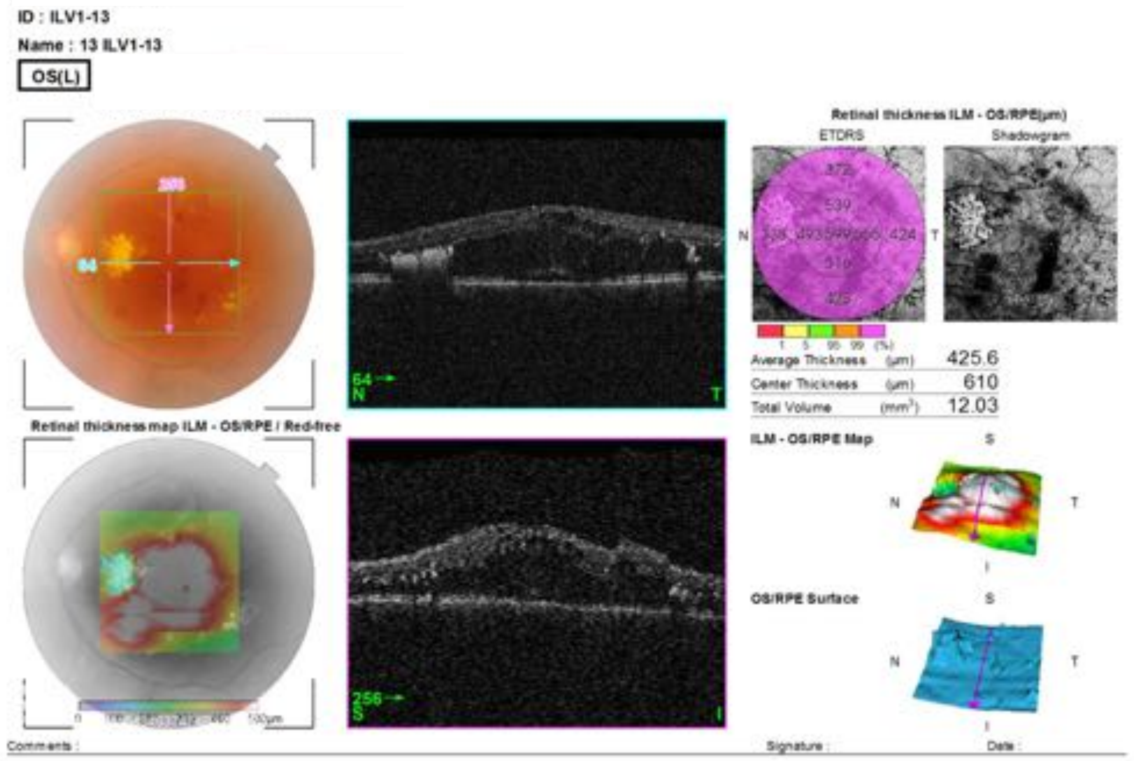

Supplement: S1 File — (PDF) [file pone.0248439.s002.pdf]
