# Supplementary material for: Vitreous expression of cytokines and growth factors in patients with diabetic retinopathy—An investigation of their expression based on clinical diabetic retinopathy grade
Source: PLoS One. 2021 May 19;16(5):e0248439. doi: 10.1371/journal.pone.0248439 (PMC8133486; doi:10.1371/journal.pone.0248439)
Supplement: S2 File — (PDF) [file pone.0248439.s003.pdf]

Flex sets CBA Fa. BD	Source	Cat. No.	Kit Lot. Nr.
Human IL-6 CBA Flex set	Becton Dickinson	558276	9275025
Human VEGF CBA Flex set	Becton Dickinson	558336	9232667
Human IL-8 CBA Flex set	Becton Dickinson	558277	101826
Human MCP-1 CBA Flex set	Becton Dickinson	558287	9363487
Human IP-10 CBA Flex set	Becton Dickinson	558280	9326136
Human CD54/ ICAM-1 CBA Flex set	Becton Dickinson	560269	8355856
Human IL-10 CBA Flex set	Becton Dickinson	558274	9263316
Human TNF CBA Flex set	Becton Dickinson	560112	9213241
Human IFN gamma CBA Flex set	Becton Dickinson	558269	9263333

ELISA-Kits	Source	Cat. No.	Kit Lot. Nr.
Human PIGF	R&D	DPG00	P238955
Human Serpin F1 /PEDF Duoset	R&D	DY1177-05	P199635
Human Erythropoetin EPO	R&D	DEPRU0	P250497
Human IL-16	R&D	D1600	P245256
